# Expansion and Conservation of Biosynthetic Gene Clusters in Pathogenic *Pyrenophora* spp.

**DOI:** 10.3390/toxins12040242

**Published:** 2020-04-09

**Authors:** Paula M. Moolhuijzen, Mariano Jordi Muria-Gonzalez, Robert Syme, Catherine Rawlinson, Pao Theen See, Caroline S. Moffat, Simon R. Ellwood

**Affiliations:** 1 Centre for Crop Disease and Management, Department of Environment and Agriculture, Curtin University, Bentley, WA 6102, Australia; 2 Canadian Centre for Computational Genomics, McGill University and Genome Quebec Innovation Center, Montréal, QC H3A 0G1, Canada

**Keywords:** necrotrophic fungal pathogen, synteny, comparative genomics, PKS, NRPS, secondary metabolism

## Abstract

*Pyrenophora* is a fungal genus responsible for a number of major cereal diseases. Although fungi produce many specialised or secondary metabolites for defence and interacting with the surrounding environment, the repertoire of specialised metabolites (SM) within *Pyrenophora* pathogenic species remains mostly uncharted. In this study, an in-depth comparative analysis of the *P. teres* f. *teres*, *P teres* f. *maculata* and *P. tritici-repentis* potential to produce SMs, based on in silico predicted biosynthetic gene clusters (BGCs), was conducted using genome assemblies from PacBio DNA reads. Conservation of BGCs between the *Pyrenophora* species included type I polyketide synthases, terpene synthases and the first reporting of a type III polyketide synthase in *P teres* f. *maculata*. *P. teres* isolates exhibited substantial expansion of non-ribosomal peptide synthases relative to *P. tritici-repentis*, hallmarked by the presence of tailoring cis-acting nitrogen methyltransferase domains. *P. teres* isolates also possessed unique non-ribosomal peptide synthase (NRPS)-indole and indole BGCs, while a *P. tritici-repentis* phytotoxin BGC for triticone production was absent in *P. teres*. These differences highlight diversification between the pathogens that reflects their different evolutionary histories, host adaption and lifestyles.

## 1. Introduction

Filamentous fungi are prolific producers of secondary or specialised metabolites (SM), which are products of metabolic pathways that have been selected for the ecological adaptation of an organism to a particular niche. Primary metabolites, in contrast, are considered essential for fungal growth, development and reproduction. SMs are not only important for survival in competitive fungal niches but can contribute to plant virulence [1,2]. The well-known polyketide T-toxin is an example of a virulence factor produced by the maize pathogen *Cochliobolus heterostrophus* that is responsible for significant crop losses [3].

Genes involved in biosynthesis, regulation and transport of SMs are commonly found in clusters and are referred to as biosynthetic gene clusters (BGCs) [4], which are categorised by specific “backbone” or “signature” enzymes and precursors involved in their biosynthesis. The major classes of compounds are polyketides, non-ribosomal peptides and terpenes, which are produced by polyketide synthases (PKSs), non-ribosomal peptide synthases (NRPSs) and terpene synthases, respectively, in conjunction with other “tailoring” or “decorating” enzymes which modify the core structure. These can include oxidoreductases, methyltransferases, acyltransferases and glycosyltransferases [5]. The occurrence of hybrid BGCs, such as PKS-NRPSs, are common where two types of biosynthetic enzymes are encoded or a hybrid enzyme containing signature biosynthetic domains from two different classes are found [6,7].

The types of signature enzyme(s) present in a genome can be identified by the active domains that catalyse the chemical biosynthesis of the different classes of SMs. As the signature domains tend to be well conserved, the major biosynthetic signature enzyme types in the genome can be identified computationally from sequence similarity searches [8,9]. For instance, NRPSs are characterised by the presence of adenylation (A), peptidyl carrier (PCP or T) and condensation (C) domains. While PKSs have distinctive ketosynthase (KS), acyltransferase (AT), acyl carrier protein domain(s) and optional reducing domains, keto reductase (KR), enoyl reductase (ER) and dehydratase (DH). These domains have sequence homology between organisms [10].

The fungal genus *Pyrenophora* contains two major crop necrotrophic pathogen species *Pyrenophora tritici-repentis* (Ptr) and *Pyrenophora teres*, the causal agents of wheat tan spot and barley net blotch, respectively. *P. teres* has two forms with similar morphologies but with distinct disease symptoms [11,12]. The two forms are largely genetically autonomous, although rare hybridisation events can occur [13,14,15,16]. In barley, *P. teres* f. *teres* (Ptt) causes net form net blotch (NFNB) and *P. teres* f. *maculata* (Ptm) spot form net blotch (SFNB). Although the full repertoire of SM compounds within *Pyrenophora* pathogenic species remains mostly unknown, previous studies in Ptr have proposed that an uncharacterised low-molecular-weight molecule (designated as ToxC) is responsible for the characteristic leaf chlorosis disease symptom during infection [17]. In addition, it has been shown that Ptr produces several anthraquinone mycotoxins, such as catenarin and emodin [18,19] and phytotoxic compounds known as triticones or spirostaphylotrichins [20,21]. *P. teres* spp. also produce several known types of compounds with phytotoxic or cytotoxic properties. The most relevant for plant disease are the non-ribosomal peptides, aspergilomarasmine and its derivatives, which showed some correlation between cultivar sensitivity and susceptibility to disease [22]. Other compounds isolated from *P. teres* include pyrenolides [23,24,25], pyrenoline [26] and also catenarin [27].

Previous PacBio genome sequencing projects for Ptr, Ptt and Ptm [28,29,30,31,32] have provided an opportunity to explore the genetic capacity of these taxa to produce specialised metabolites and explore gene cluster conservation within the three different species. To date, whole genome-based comparative investigation into specialised metabolite BGCs in Ptr, Ptt and Ptm has not been undertaken. Initial investigations into Ptr found that not all backbone biosynthetic genes were conserved between the different races of Ptr [28,29]. In Ptt, a genome expansion of low complexity (low GC) regions and NRPS genes was identified when comparatively analysed to Ptm and Ptr, and many predicted NRPSs were found to be non-canonical, with some of their main enzymatic domains missing (e.g., adenylation, peptidyl carrier protein, condensation and/or thioesterase domains) [30]. This study investigates the genome SM landscape for three important fungal diseases that have a direct negative impact on global barley and wheat yields. The genomes were analysed both between and within species to identify BGC regions that may contribute to the different pathosystems, and further investigates the apparent expansion of NRPS BGCs within *Pyrenophora*.

## 2. Results

### 2.1. PacBio Assembly Statistics for Ptt HRS09122 and HRS09139

Two new Australian Ptt isolates with differing pathotypes were sequenced to add to existing genome assemblies for four pathotypes [30] (Table 1). As Ptt has a comparatively large and variable genome size compared to Ptm and Ptr, these isolates were included to increase the potential to detect isolate-specific differences in BGCs. The total genomic assembly sizes for HRS09122 and HRS09139 were 47 Mb and 50 Mb, respectively. Furthermore, approximately 80% of the final assembly for HRS09122 was contained within the largest 11 contigs and for HRS09139 contained within the largest 12 contigs. When compared to the estimated average Ptt genome assembly size (49Mb) [30], HRS09122 and HRS09139 assemblies were approximately 1 Mb smaller and 2 Mb larger, respectively. The assembled and annotated genomes were deposited in NCBI GenBank under accessions WJSL00000000 and WJSK00000000. 

### 2.2. Genome-Wide Phylogenetic Analysis for Ptt, Ptm and Ptr

**Figure 1:**
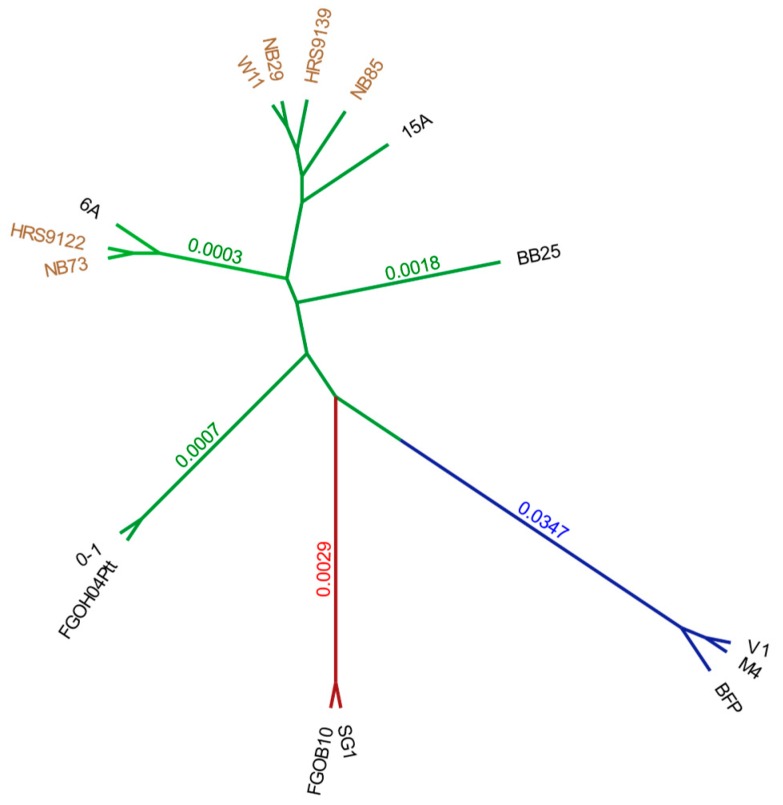
*Pyrenophora* whole genome phylogenetic analysis displays three distinct groups for *P. teres* f*. maculata* (Ptm) (red), *P. tritici-repentis* (Ptr) (blue) and *P. teres* f. *teres* (Ptt) (green). The phylogenetic tree shows the raw branch lengths on a transformed radial tree for Ptm isolates SG1 and FGOB10, Ptr isolates Pt-1C-BFP (BFP), M4 and V1, and Ptt isolates FGOH04, 0-1, NB73, HRS09122, 6A, W1-1, NB29, HRS09139, NB85, 15A and BB25. Australian Ptt isolates are labelled in brown.

A whole genome phylogenetic analysis of isolates produced distinct phylogenetic groups for the three *Pyrenophora* pathogens. All eleven Ptt isolates FGOH04, 0-1, NB73, HRS09122, 6A, W1-1, NB29, HRS09139, NB85, 15A and BB35, Ptm isolates SG1 and FGOB10, and Ptr isolates Pt-1C-BFP (BFP), M4 and V1, were clearly separated with raw branch lengths less than 0.0018, 0.0024 and 0.0002, respectively (Figure 1). Australian Ptt isolates, depicted in brown, form two phylogenetic clusters, each with a USA outgroup. The first cluster of Australian isolates (W1-1, NB29, HRS09139 and NB85) grouped with Californian isolate 15A while the second cluster (HRS09122 and NB73) grouped with Californian isolate 6A. Ptt isolate BB25 (from Denmark) was distant to both Australian clusters on a single branch. Isolates 0-1 (from Canada) and FGOH04 (from North Dakota) were furthest from the Australian isolates (Figure 1). Ptr and Ptm isolates, also with representatives from Australia and the USA, showed less branching, however, overall, the phylogenetic results indicated a broad representation of pathogen genotypes.

### 2.3. Genome-Wide Alignments Between Ptt, Ptm and Ptr

**Figure 2:**
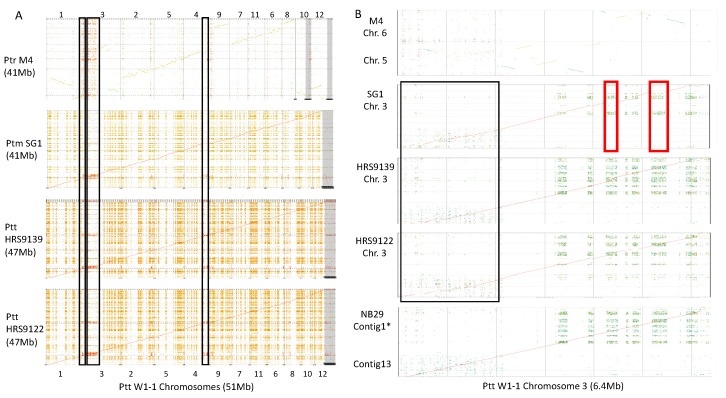
**(A**) Whole genome alignments to Ptt W1-1 (horizontal axis) for Ptt HR0S9122 and HRS09139, Ptm SG1 and Ptr M4 (vertical axis). A large conserved 2 Mb repeat region in *P. teres* and Ptr chromosomes was identified on W1-1 chromosome 3, and smaller regions were also found conserved to the distal regions of W1-1 chromosomes 1 and 4 (boxed in black). (**B**) Chromosome 3 alignments between Ptt W1-1 (horizontal axis) and Ptt NB29, HRS09122, HRS09139, Ptm SG1 and Ptr M4 chromosomes 5 and 6 (vertical axis). Deletion sites within the large 2 Mb chromosome 3 repeat region were observed in Ptt HRS09122, HRS09139 and Ptm SG1 (boxed in black), while Ptt NB29 was highly colinear to W1-1 within this region. Ptr M4 had reduced alignments to W1-1 chromosome 3. The absence of low complexity regions in Ptm are highlighted in red, which are the regions of expansion in Ptt.

The genomes of all isolates were aligned to Ptt isolate W1-1 (the largest Pyrenophora genome). Co-linear alignments were observed between whole chromosomes for Ptt and Ptm with large-scale rearrangements between *P. teres* and Ptr. An absence of low complexity regions in Ptm relative to Ptt was evident which represent regions of expansion in Ptt as previously reported [30] (Figure 2). For W1-1, a large 2 Mb repeat region (chr3:1-2,000,000) and smaller regions in chromosome 1 and 4 subtelomeres were clearly conserved to Ptt, Ptm and Ptr isolates (Figure 2A). However, for Ptm isolate SG1 and Ptt isolates HRS09122 and HRS09139 large deletions were observed in the 2 Mb repeat region of chromosome 3, while Ptt isolate NB29 was more highly conserved to W1-1 (Figure 2B).

### 2.4. Pyrenophora BGC in Silico Prediction

**Figure 3:**
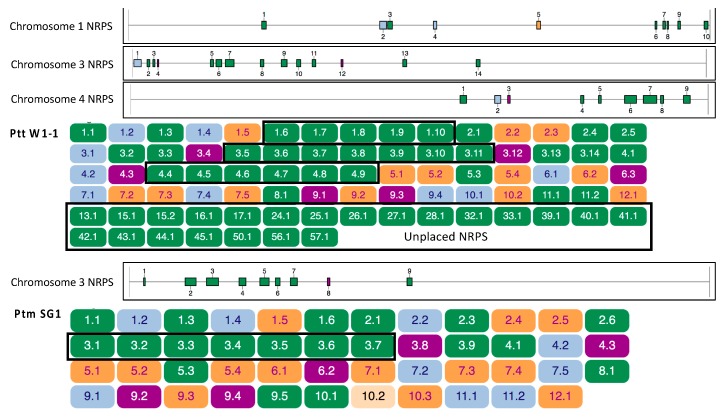
Overview of the predicted biosynthetic gene clusters (BGCs) for *P. teres* f. *teres* isolate W1-1 (top) and *P. teres f*. *maculata* isolate SG1 (bottom). The non-ribosomal peptide synthase (NRPS) expansion and locations for W1-1 chromosomes 1, 3 and 4, and SG1 chromosome 3 are boxed in black. The majority of W1-1 NRPS biosynthetic gene clusters were situated on assembled chromosomes, with a large number on unplaced contigs. BGCs are colour coded as green for NRPS, orange for polyketide synthase (PKS), light blue for NRPS-PKS hybrids and purple for terpenes. Each cluster is labelled by chromosome or contig number and by cluster number (chromosome or contig.cluster). Relative locations of BGCs across chromosomes are shown above BGC predictions.

The search for specialised metabolite BGCs in Ptr, Ptm and Ptt genomes identified NRPS, NRPS-like, type I PKS (T1PKS), type III PKS (T3PKS), hybrid T1PKS-NRPS and terpene predictions in all isolates. The total number of predictions for Ptt W1-1, Ptm SG1 and Ptr M4 were 82, 47 and 39, respectively (Table S1). The type of BGCs found in each isolate based on the signature biosynthetic enzymes encoded are presented in Table 2. The number of BGC for each genome varied including the number of clusters for each type, however only a single, orthologous, T3PKS was found in all isolates. The majority of predicted BGCs were NRPS and T1PKS in all genomes. While Ptt had the largest variance in NRPSs numbers, which ranged between 29 and 53, as compared to 15 - 16 and 10 - 13 for Ptm and Ptr, respectively. A single indole containing BGC was predicted for Ptm and a single hybrid NRPS-indole BGC was found in all Ptt isolates, while no indole BGCs were predicted in Ptr. The number of NRPS regions identified in Ptt was on average approximately four times that of Ptr and over twice that of Ptm (Table 2). Many Ptt NRPSs were located on chromosomes 1, 3 and 4, which coincided with repeat regions. However, Ptt NRPSs were also highly represented in chromosomally unplaced fragmented contigs, which commonly occur in highly repetitive regions (Figure 3). Many of such regions appeared to lack the order and domains necessary for complete polyketide or non-ribosomal peptide synthases. The predicted BGCs were positioned throughout genome chromosomes, with many NRPS and PKS clusters in close proximity to distal and subtelomeric regions in Ptt and Ptm (Figure S1). Over half of Ptt W1-1 predicted BGCs that contained NRPS modules (NRPS, T1PKS-NRPS hybrid, NRPS-like and NRPS-indole), a total of 41 of the 63, were located on assembled chromosomes while the remainder were located on unplaced contigs (Table S1). A disproportionate number NRPS containing BGCs, a total of 12, were located on chromosome 3. The chromosome 3 NRPSs were co-located with quantitative trait loci (QTLs) associated with virulence [33]. While the majority of Ptm NRPS containing BGCs were also located on chromosome 3 (7 BGCs), a total of 17 NRPS BGCs were distributed across five Ptr M4 chromosomes (Figure S1).

### 2.5. Orthologous Clustering of NRPS Backbone Protein Sequences

Across all isolates, a total of 583 NRPS genes were clustered and 567 (97.3%) were assigned to 25 orthologous groups. Three orthologous groups were present in all isolates and one of these consisted entirely of single-copy genes. Ten groups were unique to Ptt and no groups were Ptr or Ptm specific. Clustering NRPS analysis found up to 13 and 2 gene duplication events for Ptt and Ptr, and none were found for Ptm isolates FGHB10 and SG1 (Table S2).

### 2.6. Orthologous Clustering of T1PKS Backbone Protein Sequences

Across all isolates, 264 predicted T1PKS genes (100%) were assigned to 23 orthologous groups and of these only four orthologous groups were found core to Pyrenophora represented by all isolates. Orthologous groups were also identified unique and core to a species, represented by all the species isolates, these included four groups core specifically to Ptr and four groups core specifically to *P. teres*. No orthologous groups were core uniquely to Ptm alone. Only one group contained gene duplication events for Ptr Pt-1C-BFP, M4 and V1. The remaining Ptt and Ptm T1PKS were all single copy genes (Table S2).

### 2.7. Biosynthetic Gene Duplications in Pyrenophora

**Figure 4:**
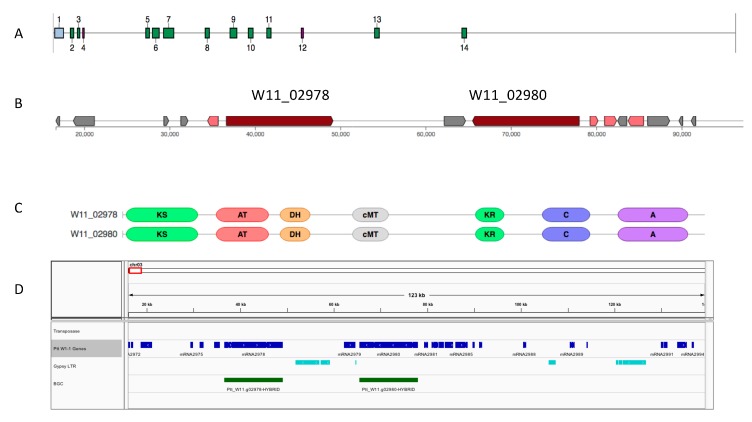
Ptt isolate W1-1 gene duplication event in a predicted hybrid NRPS-T1PKS biosynthetic gene cluster region on chromosome 3 (16,646–98,007 bp) (BGC 3.1). (**A**) W1-1 chromosome 3 biosynthetic cluster locations, NRPS (green) and NRPS-T1PKS (blue). (**B**) Gene content for the hybrid NRPS-T1PKS biosynthetic gene cluster, backbone genes (red) and additional biosynthetic genes (pink). (**C**) Domain structures for the backbone BGC genes W11_2978 and W11_02980. (**D**) Genomic location (chr03:16,000–140,000 bp) for biosynthetic gene cluster core duplicated genes (green) and Gypsy long terminal repeat (LTR) fragments (light blue), with all genes are shown in blue.

In the regions of biosynthetic cluster expansions, clear gene and regional duplications were found involving signature biosynthetic genes. One such example involved a gene duplication event for a W1-1 predicted hybrid NRPS-T1PKS (BGC region 3.1) with a subtelomere position on chromosome 3 (chr3:16,646 - 98,007 bp) (Figure 4).

**Figure 5:**
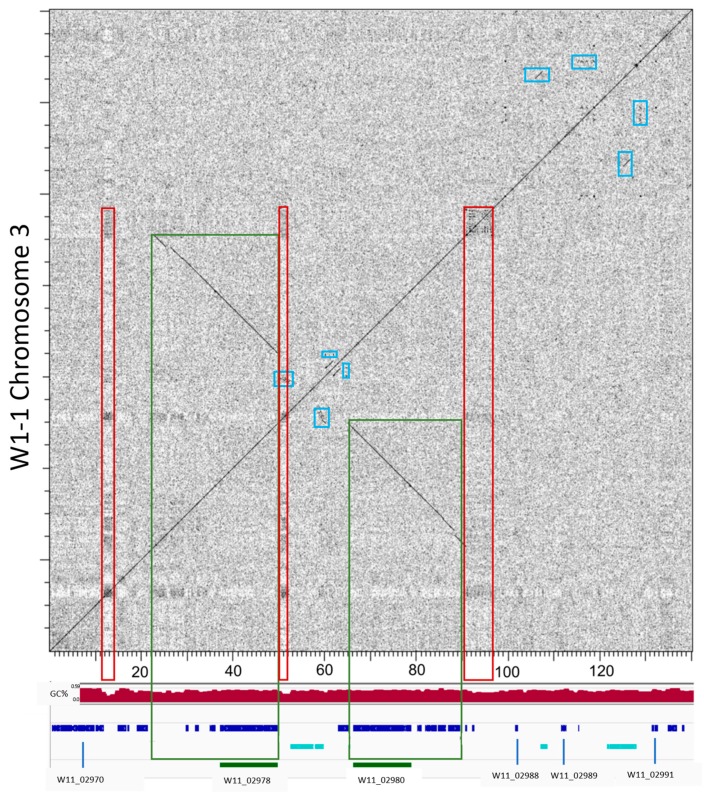
*P. teres f. teres* (W1-1) chromosome 3 subtelomere (1–140,000 bp) has backbone biosynthetic gene duplications flanked by LTR Gypsy fragments and ancient sites of thioesterase (TE) tandem inverted repeats (TIRs). The nucleotide sequence plot (above) shows the biosynthetic backbone duplicated gene regions (boxed green), ancient sites of tandem repeats and LTR fragments (boxed blue) and low GC regions (boxed red). The plotted region (below) shows sequence percentage GC (window size 1kb) (red graph), and the locations of genes (blue), LTR gypsy fragments (light blue) and the duplicated backbone genes (green).

Closer examination of W1-1 chromosome 3 subtelomere sequence (1–140,000 bp) found that the core biosynthetic gene duplications were flanked by long terminal repeat (LTR) Gypsy elements and ancient sites of inverted tandem repeats (TIR) (Figure 5).

### 2.8. Pyrenophora teres Regions of NRPS Expansion are Subtelomeric

**Figure 6:**
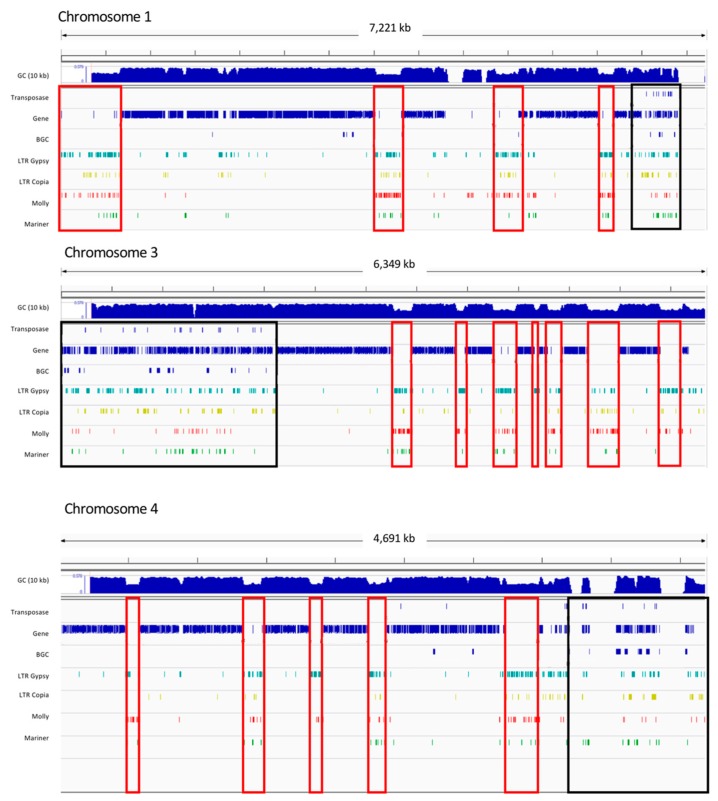
Distinct regions of NRPS expansion associated with the subtelomeric regions of chromosomes 1, 3 and 4 in Ptt isolate W1-1. NRPS biosynthetic gene cluster expansion regions in GC equilibrated regions are interspersed with transposable elements (boxed in black). Regions of low complexity (boxed in red) are packed with LTR Copia (yellow) and Gypsy (green) elements, DNA/TcMar-Fol1 molly (red) and Mariner elements (light green).

On closer examination of the W1-1 NRPS gene expansions, distinct GC equilibrated regions of NRPS duplications were associated with the subtelomeric regions of chromosomes 1, 3 and 4. The largest (~2 Mb on chromosome 3) was conserved in Ptm but absent in Ptr. Class I LTR retrotransposon (Copia and Gypsy) elements and Class II DNA transposons (Mariner) elements, that were confined to low GC repeat regions in Ptt, were co-located with the GC equilibrated NRPS gene expansions on chromosomes 1, 3 and 4, a probable result of ancient transposable element activity (Figure 6).

### 2.9. Comparative Analysis of the Predicted BGCs in Pyrenophora

**Figure 7:**
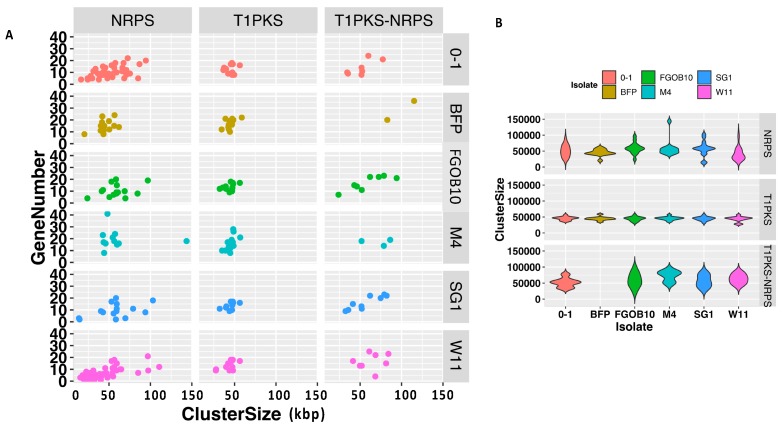
Predicted biosynthetic gene cluster sizes and gene numbers were compared for Ptm, Ptt and Ptr. (**A**) NRPS, T1PKS and T1PKS-NRPS BGCs cluster size versus gene number for Ptm isolates FGOB10 and SG1, Ptr isolates M4 and Pt-1C-BFP (BFP) and Ptt isolates 0-1 and W1-1. (**B**) Violin plot of NRPS, T1PKS and T1PKS-NRPS BGCs cluster sizes.

The size and gene content of BGCs were compared for all isolates. Predicted BGC types for representative isolates from Australia and America were selected for Figure 7. Ptr M4 had the largest NRPS cluster size (143 kb) (Chr3: 1,105,219-1,515,924) and the largest NRPS cluster of genes (41 genes) (Chr5:179,450-227,625). The number of NRPS-T1-PKS hybrids was notably less in Ptr, two and three for Pt-1C-BFP and M4, respectively, compared to Ptt and Ptm which ranged between six and nine BGC regions. The only exception was Ptt BB25 which had two hybrid clusters. The number of BGC genes and cluster sizes for hybrid NRPS-T1-PKSs were consistent, with Ptr Pt-1C-BFP as an exception, which had the largest number of genes (36 in total) and cluster size (115 kb) (contig4:685,279-800,416). T1PKS BGCs were consistent in the number of genes and cluster size ranges across Ptr, Ptm and Ptt (Figure 7).

### 2.10. Biosynthetic Gene Clusters Conserved in Pyrenophora

Conservation of BGCs between *Pyrenophora* species was determined relative to Ptr M4. Out of the 39 predicted Ptr M4 BGCs, a total of 13 BGCs are conserved in either Ptm SG1 or Ptt W1-1. These include eight NRPSs, eight T1PKSs, one T3PKS, three terpenes, three NRPS-like and one NRPS-T1PKS cluster. A total of 14 BGCs were conserved in *P. teres* and not with Ptr (Table S3). There are six conserved biosynthetic gene clusters across the three pathogens with annotated potential products, alternapyrone (T1PKS), apicidin (NRPS), betaenone (T1PKS), dimethylcoprogen (NRPS), melanin (T1PKS) and pestheic acid (T1PKS). A PKS-NRPS hybrid biosynthetic gene cluster (Ttc) responsible for triticone production [21] was present in all Ptr isolates and absent in all Ptt and Ptm isolates. This cluster, located on Ptr M4 chromosome 4, has known conservation with *Curvularia pallescens* an anamorph (asexual stage) of *Cochliobolus pallescens* [21] (Figure S2). A total of 13 BGCs for *Pyrenophora* have a potential product based on similarity to characterised BGCs and can be found in Table 3.

### 2.11. NRPS Module Domain Nitrogen Methyltransferase Distinguishes P. Teres

**Figure 8:**
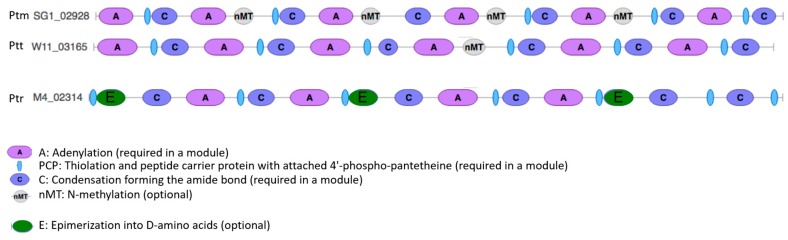
NRPS nitrogen methyltransferase domains distinguishes *P. teres* from *P. tritici-repentis.* Nitrogen methyltransferase (nMT), adenylation (A), condensation (C) and epimerization (E) domains are shown for Ptm (SG1_02928), Ptt (W11_03165) and Ptr (M4_02314) NRPS genes.

The order of modules and domains of a complete non-ribosomal peptide synthetase require an initiation or starting module ([F/NMT]-A-PCP-), elongation or extending modules (-(C/Cy)-[NMT]-A-PCP-[E]-) and a termination or releasing module (TE/R). Only one region (region 42.1) in W1-1 had a termination by a thioesterase (TE) on a fragmented unplaced contig. On closer examination of the *P. teres* predicted NRPS methylation domains (MT), a bias for tailoring cis-acting nitrogen methyltransferase (nMT) enzyme domains was identified. A total of 30 of the 49 NRPS BGCs had nMT domains represented and only a single carbon methyltransferase (cMT) domain (W11_10429) was identified for a NRPS BGC. However, for Ptr, only a single nMT domain containing NRPS BGC (M4_07432) and no cMT domain containing NRPS BGCs were identified. While for Ptm (SG1), 5 out of 19 NRPS BGCs were nMT domain containing and all hybrid NRPS-T1PKS signature genes were cMT domain containing BGCs. In contrast, Ptr had more NRPS modules represented by epimerization into D-amino acid domains than *P. teres* (Figure 8).

The number of nMT domains were searched by tblastn [34] at greater than 50% sequence identity and greater than 80% domain coverage in all available genomes and only a single gene domain was represented in all Ptr isolates (Table 4).

## 3. Discussion

### 3.1. Pyrenophora Whole Genome Comparative Analysis

The whole genome phylogenetic analysis of *Pyrenophora* isolates showed that Ptt isolates from Australia were similar to two groups of isolates from California. California shares a similar Mediterranean climate to many of the barley growing regions of Australia and selection of cultivars adapted to a Mediterranean climate by breeders may influence genotype distribution as they may be preferred hosts for particular pathotypes. As Ptt is dispersed on infected seed, phylogenetic similarities may also be related to cultivation of historic cultivars, for example the American cultivar Beecher in Western Australia. Alternatively, as the genotypes in Australia appear to represent a subset of USA and European diversity, and with recent population studies confirming two groups occur in Australia [33,35], the genotypes present in Australia may represent chance incursions.

Overall, although there is a clear bias in the number of isolates in this study towards Ptt, this pathogen showed deep phylogenetic branching between isolates. This is consistent with the degree of host specialization, genome size and the length of coexistence of each of these species with their respective hosts, discussed in Syme et al. (2018) [30]. Ptm and Ptr only became significant pathogens within the last 100 years [36,37,38,39,40]. Ptt, by contrast, has a lengthy association with barley that may go beyond the earliest written records of leaf diseases. Ptt produces both necrotrophic effectors, characteristic of pure necrotrophs, and avirulence genes, characteristic of biotrophs, and is notable for complex host-pathogen genetic interactions [12,41,42] compared to Ptr, where three main effectors explain most of the disease [43], and Ptm where minor effect QTL condition disease [44]. In general, the phylogeny presented supports the diversification of Ptt and recent divergence of Ptm and Ptr [11,30].

### 3.2. Specialised Metabolites in Pyrenophora

This is the first comparative analysis of *Pyrenophora* specialised metabolites, based on whole predicted biosynthetic gene cluster regions for three pathogenic *Pyrenophora* species. A suite of NRPS, T1PKS, T3PKS and hybrid BGCs were identified for 17 isolates with PacBio genome assemblies. The majority of BGCs were represented by NRPSs and Ptt had a greater than expected number as indicated in a previous study for this barley pathogen [30]. Although T3PKS have been studied in Ptr and Ptt [45] we established for the first time that this predicted gene cluster is highly conserved between the three *Pyrenophora* species. T3PKS are distinct from iterative T1PKS as they are independent of acyl carrier protein (ACP) and act directly on acetyl CoA substrates [46].

Very few predicted BGCs had predicted structures similar to previously characterised biosynthetic clusters. The most conserved BGC in Ptm, Ptr and Ptt shares 100% gene similarity with the known alternapyrone BGC (Table S2), a small five gene biosynthetic cluster which was first isolated from *Alternaria solani*, the causal agent of early blight disease in tomato and potato [47]. Other specialised phytotoxic metabolites in *P. teres* have been identified that correlate with late disease symptoms, which include isoquinoline, pyrenolines, pyrenolides and the peptide alkaloids aspergilomarasmine and its derivatives as reviewed by Muria-Gonzalez et al. in 2015 [48]. However, identification of the BGCs synthesising these products have yet to be characterised. Although there was clear conservation within *Pyrenophora,* there was low overall BGC conservation with known BGCs in other species. This is consistent with less conservation of non-core BGCs genes between species, while it is common that backbone biosynthetic genes are conserved.

### 3.3. Absence of Triticone BGC in P. teres Forms

Although a number of Ptr BGCs regions appeared conserved in *P. teres*, a BGC involved in production of a phytotoxic triticone [21] was absent in *P. teres* but highly conserved with more distant *Pleosporaceae* species such as *Staphylotrichum coccosporum*, *Curvularia pallescens*, *Cochliobolus*/*Bipolaris* spp. and *P. seminiperda* [21]. This type of phenomenon was also observed for a necrotrophic effector gene, *ToxA*. Though a 12 kb homologous region containing the *ToxA* gene was present in Ptr and other distantly related *Pleosporaceae* species including *Parastagonospora nodorum* and *Bipolaris sorokiniana* [49,50,51], this effector gene is absent in the genomes of *P. teres*. A recent horizontal gene transfer (HGT) event led to the acquisition of the *ToxA* gene in Ptr and *B. sorokiniana*, although the origin of the donor is unknown. The HGT of *ToxA* demonstrated that genes can be horizontally acquired across multiple species that benefits the lifestyle of the fungal species, i.e., increase in virulence. The BGC involved in the production of the phytotoxic triticone is conserved in Ptr, however disruption of triticone production did not affect the vegetative growth or the ability of the Ptr pathogen to infect the host [21]. Nevertheless, Ptr triticone exhibits mild antibacterial property and was therefore proposed to provide an advantage in multi-microbial environments. The absence of the triticone BGC in *P. teres* may thus reflect a lifestyle distinction between Ptr and *P. teres* which does not involve virulence on a host. The evolutionary history of this gene cluster in *Pyrenophora* is uncertain, possibly being lost in the *P. teres* lineage or acquired via a HGT event in Ptr, similar to the *ToxA* [36].

### 3.4. NRPS Gene Expansion Association with Transposable Elements in P. teres

Ptt has a larger and more repetitive genome than Ptm and Ptr, with frequent large (20–40 kb) stretches of AT-rich regions, which may correlate with a longer association with a cultivated host compared to Ptm and Ptr [30]. Although macro-synteny of Ptt gene-rich or GC equilibrated regions has been shown between Ptt and Ptm, and an expanded array on NRPS BGCs was reported in Ptt compared to Ptm [30]. Comparative analysis of the predicted BGCs based on the latest version of fungi-smash in this study identified a larger number of NRPS regions than previously reported. Furthermore, these NRPS expanded regions were identified on chromosomes 1, 3 and 4 in Ptt and were conserved between Ptt and Ptm on chromosome 3.

In *P. teres* isolates, distinct GC equilibrated regions of expansion were associated with Class I and II transposable repeat elements, which are usually confined (in high density) to the low GC repeat regions. In the NRPS GC equilibrated regions, gene duplications and ancient transposase activity were evident and far more prominent in Ptt than in Ptm. The NRPS expansion showed an invasion of Class 1 retrotransposons, (LTR Copia and Gypsy elements) and Class II DNA transposons (Mariner type) that are ubiquitous in plant genomes, and may represent horizontal transfers during infection [52]. Transposable elements (TEs) are known not to be randomly distributed, are extensively modified through RIP mutations [53], and are associated with genome rearrangements and horizontal gene transfers between different species [54,55]. TEs can also contribute cis-regulatory DNA elements to the modification of transcriptional networks [55]. Co-located TEs could plausibly have contributed to the expanded arrays of NRPS BGCs. Furthermore, during meiosis, chromosome breakage fusion cycles begin with the loss of telomeres which causes the instability of the distal regions, and potential fusion of sister chromatids [56]. The site of breakage during separation in erroneously fused sister chromatids can lead to sequence duplication, deletion and rearrangement [56,57,58]. Therefore, breakage fusion events may have also contributed to NPRS duplication in Ptt.

### 3.5. Are the Predicted Biosynthetic Gene Clusters in P. teres Complete?

The genomes in this study were sequenced by long-read PacBio chemistry, with an average read length of 10 kb, which efficiently capture gene-rich regions and low complexity regions. However, in regions where repetitive sequences are large, the assembly of segmental duplications may fragment, but gene content would still be captured. Large scale chromosomal structural variations caused by breakage and fusion events could also destabilize gene clusters. Many of the Ptt NRPS BGCs appeared incomplete and located on unplaced fragmented contigs which may be interrupted by such regions. However, short regions containing BGC genes, to complement incomplete BGCs, were not evident. The absence of the necessary modules for the core genes suggest that many of these BGCs may not be functional, and require experimental validation.

### 3.6. Pyrenophora Species Have Different Tailoring Enzyme Domains

The expansion in *P. teres* NRPSs was hallmarked by the presence of tailoring cis-acting nitrogen methyltransferase (MT) domains as compared to Ptr. BGCs have a number of different tailoring enzymes that catalyse a variety of chemical modifications to the central SM backbone which enhance structural diversity. MT domains are present in both NRPS and PKS clusters. MTs catalyse the methyl transfer from S-adenosylmethionine (SAM or AdoMet) to the carbon, nitrogen or oxygen atoms at various positions on the backbones of polyketides, non-ribosomal peptides and fatty acids and are therefore classified as cMT, nMT and oMT, respectively, depending upon their site of methylation. *P. teres* NRPSs have a clear bias for cis-acting nMT domains compared to Ptr with no co-occurrence of different MT types within these BGCs. An HMM profile search of NCBI nr found that in most cases proteins had oMT domains and relatively few cMT and nMT domains [59]. In contrast to oMTs, the nMT and cMT domains are present in multifunctional enzymes with other catalytic domains in addition to those found in NRPS gene clusters. *P. teres* may therefore potentially produce secondary metabolites with a variety of structural modifications. Furthermore, the overrepresention and role of Ptr NRPS D-amino acid epimerization domains remains to be explored.

Functional assays are required to investigate the physiological role of individual nMT domains in *P. teres*. nMTs are involved in a wide variety of processes but may have specific functions in providing small molecules for specialized pathways. These pathways may relate to adaption to barley as a host, or to saprophytic stages outside of the barley growing season, where *P. teres* competes with other fungi. *P. teres* species are regarded as hemibiotrophs and have adapted intimately to barley [30] as opposed to the necrotrophic pathogen Ptr which is intimately adapted to wheat [60]. It is therefore possible that the tailoring enzyme differences between Ptr and *P. teres* may be correlated with their phytopathogen class and host specialisation [30].

## 4. Conclusions

This study provides a comprehensive comparative analysis into potential metabolic variation of three *Pyrenophora* pathogens, based on in silico biosynthetic gene clusters, which highlighted their diversification. Secondary metabolite biosynthetic capacity of the *Pyrenophora* pathogens was determined from high quality PacBio assemblies and illustrated that Ptt has the highest potential to produce large numbers of different specialised metabolites. A relative NRPS expansion in Ptt but to a lesser degree in Ptm and absence in Ptr may correlate intrinsically with differing genome architecture and length of time since disease emergence in cultivation. The NRPS expansion in Ptt may therefore reflect greater host specialisation.

Although conservation of BGCs was found between the *Pyrenophora* species, which included the first reporting of a type III polyketide synthase BGC in Ptm, differences highlighted diversification that could support their different evolutionary histories and host-pathogen interactions. It is envisioned that this analysis will contribute towards specialised metabolite gene cluster characterisation to inform future analyses on specialised metabolite diversity within *Pyrenophora*.

## 5. Materials and Methods

### 5.1. Isolates Sequenced in This Study

Two new Australian Ptt isolates, HRS09122 and HRS09139, from New South Wales and South Australia, respectively, were sequenced by PacBio chemistry. Isolate HR09122 displays high virulence on barley cultivars Skiff and Gilbert, and isolate HRS09139 displays high virulence on barley cultivar Fleet. The genomes and annotations for HRS09122 and HRS09139 have been deposited under NCBI GenBank accessions WJSL00000000.1 and WJSK00000000.1, respectively.

### 5.2. DNA Preparation and PacBio Sequencing

HRS09122 and HRS09139 DNA preparation and sequencing was performed as described by Syme et al., 2018 [30]. Briefly, fungus was grown in a low sucrose liquid culture followed by enzymatic digestion with Extralyse (Laffort, Bordeaux, France). To reduce polysaccharide contamination, a high salt CTAB procedure was used to extract the genomic DNA.

PacBio (Pacific Biosciences, Menlo Park, CA, United States) single molecule, real-time (SMRT) genome sequencing was performed on isolates at the McGill University and Génome Québec Innovation Centre (Montreal, QC, Canada) in accordance with PacBio protocols.

### 5.3. Genome Assemblies

Isolate PacBio reads were self-corrected, trimmed and assembled with Canu v1.3 [61] using an estimated error rate of 0.03, genome size 51 Mb and “pacbio-raw”. W1-1 Illumina reads [30] were mapped to the Canu assembly using BWA index and mem algorithms v0.7.15-r1140 [62] using default settings. The uniquely mapped reads were filtered using SAMtools v1.3.1 [63] and passed to Pilon v1.17 [64] to correct remaining SNP and small indel errors with default settings.

### 5.4. Specialised Metabolite Gene Cluster Identification

The genomes of Ptr isolates (Pt-1C-BFP, M4 and V1), Ptt isolates (0-1, 15A, 6A, BB25, FGOH04Ptt-21, HRS09122, HRS09139, NB29, NB73, NB85 and W1-1) and Ptm isolates (SG1 and FGOB10) were searched for predicted specialised metabolite gene cluster candidates using antiSMASH version 5.0.0 [65] using the following command line parameters, “--taxon fungi, --fullhmmer, --cassis, --cf-create-clusters, --smcog-trees, --cb-general, --cb-subclusters, --cb-knownclusters, --asf, --pfam2go”, with cluster finder probabilistic Biosynthetic Gene Clusters (BGC) detection, using minimum cluster size 5, minimum number of biosynthesis-related PFAM domains, Minimum ClusterFinder probability of 60%, known cluster and subscluster blast, smCoG analysis and active site finder (Table S1).

### 5.5. Other Pyrenophora spp. Genome Sequences Available for Analysis

The following sequenced genomes were downloaded from NCBI GenBank. Ptr race 1 isolates M4 (NQIK01000000.1) [29], V1 (SAXQ00000000) [66], Pt-1C-BFP (AAXI01000000.1) [28]. Ptt isolates included Beecher virulent W1-1 (OCTH01000000.1), Harbin virulent 0-1 (NPOS01000000.1) [31], FGOH04Ptt-21 (VBVN01000000.1), 6A (VFEN01000000.1), 15A (VBVL01000000.1) [32], Beecher virulent BB25 (VBVM01000000.1), Yeong, Maritime and Kombar virulent NB29 (WJSO01000000.1), Shepherd virulent NB73 (WJSN01000000.1) and Prior, Corvette and Gilbert virulent NB85 (WJSM01000000.1) [30]. Ptm isolates included SG1 (OCTF01000000.1) [30] and FGOB10 (PRJNA417860) (unpublished).

### 5.6. In-Silico Search for Syntenic Regions Between Pyrenophora Isolates

BGC nucleotide sequences were extracted for all the predicted specialised metabolite gene cluster genomic regions (Table S1). The genomic regions were then aligned to all the available genomes using BLAT [67], in fastMap mode at ≥ 70% sequence identity. The results were then parsed for alignments ≥ 50% sequence coverage (Ptm-Ptt ≥ 80%) and the corresponding genomic sequences were aligned and visualized using EasyFigure [68].

### 5.7. NRPS and PKS Backbone Gene Clustering

The backbone genes within NRPS and PKS clusters were extracted for all isolates and protein sequences clustered using Orthofinder version 2.3.3 [69] at an expected value ≤ 1 × 10^−10^ to identify backbone orthologous groups (orthogroups). Based on all the orthogroups, both gene and species level trees are inferred to identify gene duplication events, which are mapped to the species and gene tree locations and the percent retention of the duplicate gene in sampled species were indicated as well as genes descended from the gene duplication event [70]. This is done with Duplication-Loss-Coalescence (DLC) analysis for a more parsimonious interpretation of resolved gene trees [70]. Gene-duplication events were only considered when at least 50% of the descendant species had retained both copies of the duplicated gene.

### 5.8. Whole Genome Analysis

Genomes were aligned with MUMmer application NUCmer [71]. Genome phylogeny distances were calculated using Andi version 0.12 with Kimura model, 1000 bootstraps and 0.05 significance [72]. The distance matrices were then analysed using PHYLIP Kitsch (Fitch-Margoliash method version 3.696 with power 2.0 and for multiple datasets) and Consense version 3.696 with Majority Rule extended (MRe) and no rooted outgroup [73]. The tree was plotted with FigureTree v1.4.4 (https://github.com/rambaut/Figuretree/) with a midpoint root, raw branch lengths and as radial cladogram for visualisation.

## Figures and Tables

**Table 1:** Genome assembly statistics for two new Australian *P. teres* f. *teres* (Ptt) isolates, HRS09122 and HRS09139 compared to published genomes of Ptt isolates 0-1 and W1-1.

Isolate	^a^0-1	^b^W1-1	HRS09122	HRS09139
Collected	Canada	Western Australia	New South Wales	South Australia
Barley cultivar virulence	Harbin	Beecher	Skiff and Gilbert	Fleet
*Assembly*				
Number contigs/scaffolds*	55*	57*	44	91
Total length (Mb)	46.51	53.0	47.98	50.89
Mean size (Mb)	845.67	931.24	109.05	559.33
Median size (kb)	85.05	46.63	92.01	49
Maximum size (Mb)	5.91	7.26	5.91	6.04
Minimum size (kb)	27.93	18.74	21.64	1374
Sequences > 10 kb (%)	55(100.00)	57(100)	44(100.00)	78(85.71)
Sequences > 100 kb (%)	23(41.82)	12(21.05)	19(43.18)	23(25.27)
Sequences > 1 Mb (%)	12(21.82)	12(21.05)	15(34.09)	13(14.29)
N50 (Mb)	4.3	4.7	3.2	3.1
L50	5	5	6	6
N80	2,252,530	3,129,043	2,043,292	2,506,541
L80	10	9	12	11
*Genes*				
Protein-coding genes	11,573	11,245	10,555	10,579
NCBI/ENA BioSample	SAMN07291684	ERS1459214	SAMN13065568	SAMN13065569
NCBI locus tag	NA	PttW1-1	GD583	GD582

^a^ Published scaffolded genome [31], ^b^ published scaffolded genome version 2 [30].

**Table 2:** Number of *Pyrenophora* predicted biosynthetic gene clusters in Ptr, Ptm and Ptt genomes.

Isolates	NRPS	T1 PKS	T1 PKS-NRPS Hybrid	NRPS-Like	Terpene	T3PKS	T1 PKS-NRPS-Like	NRPS-Indole	Indole	Total
*P. triciti-repentis*										
BFP	13	12	2	4	5	1	0	0	0	37
M4	11	13	3	6	5	1	0	0	0	39
V1	10	14	2	6	5	1	1	0	0	39
*P. teres* f*. maculata*										
FGOB10	15	12	8	3	5	1	0	0	1	45
SG1	16	12	9	3	5	1	0	0	1	47
*P. teres* f. *teres*										
0-1	42	12	7	3	6	1	0	1	0	72
15A	48	11	7	3	5	1	1	1	0	77
6A	51	12	6	3	5	1	0	1	0	79
BB25	53	12	2	2	4	1	0	1	0	75
FGOH04	43	15	6	4	6	1	0	1	0	76
HRS9122	30	13	7	3	5	1	1	1	0	61
HRS9139	32	13	6	3	5	1	0	1	0	36
NB29	26	13	6	3	6	1	0	1	0	38
NB73	34	14	7	3	5	1	0	1	0	39
NB85	29	13	6	3	6	1	0	1	0	59
W1-1	49	12	8	5	6	1	0	1	0	82

**Table 3:** *Pyrenophora* biosynthetic gene clusters with potential products.

Potential Products	Ptr	Ptm	Ptt
***Conserved all pathogens***			
Alternapyrone	1	1	1
Apicidin	1	1	1
Betaenone	1	1	1
Dimethylcoprogen	1	1	1
Melanin	1	1	1
Pestheic acid	1	1	1
***Conserved P. teres***			
ACR-toxin	-	1	1
Azanigerone	-	1	1
Fusarubin	-	1	1
Phomasetin	-	1	1
***Not conserved***			
Curvupalides/Triticone (Ttc)	1	-	-
Dehydrocurvularin	1	-	-
Copalyl diphosphate	-	-	1

**Table 4:** Count of NRPS biosynthetic gene nMT domains for *Pyrenophora.*

Species	Number of Genomes Searched	Range Percent Identity	Coverage	nMt Domains Genome Count
*P. tritici-repentis*	4	66	100	1
*P. teres f. teres*	12	54–100	91–100	67–93
*P. teres f. maculata*	2	67–99	99–100	11–12
*P. serminiperda* (Illumina)	1	61	95	1
*B. maydis* (Illumina)	2	62–72	100	3
*B. cookie* (Illumina)	1	64–72	100	4
*B. sorokiniana* (Illumina)	3	67–84	100	6
*B. zeicola* (Illumina)	1	56–70	100	3

## Supplementary Materials

The following are available online at https://www.mdpi.com/2072-6651/12/4/242/s1, Table S1: Predicted *Pyrenophora* biosynthetic gene clusters for *P. teres* f*. teres, P. teres* f*. maculata* and *P. tritici-repentis* isolates with genomic locations, Figure S1: *Pyrenophora* predicted BGC distributions in PttW1-1, Ptm SG1 and Ptr M4 genomes, Table S2: Predicted NRPS and T1PKS backbone gene orthologous groups (GO) and duplication events for *P. teres* f. *teres*, *P. teres* f. *maculata* and *P. tritici-repentis* isolates, Figure S2: Biosynthetic cluster for triticone production (Ttc cluster) in *P. tritici-repentis* is absent in *P. teres f. teres* and *P. teres f. maculata.* A) Chromosome 4 *P. tritici-repentis* triticone biosynthetic gene cluster (Ttc). B) Domains for core gene M4_09010. C) Biosynthetic gene cluster conservation with *Curvularia pallescens*, Table S3: Predicted *Pyrenophora* biosynthetic gene clusters for *P. teres* f*. teres* W1-1*, P. teres* f*. maculata* SG1 that are conserved with *P. tritici-repentis* M4.
