# Supplementary material for: Expansion and Conservation of Biosynthetic Gene Clusters in Pathogenic Pyrenophora spp
Source: Toxins (Basel). 2020 Apr 9;12(4):242. doi: 10.3390/toxins12040242 (PMC7232245; doi:10.3390/toxins12040242)
Supplement: Supplementary file 1 [file toxins-12-00242-s001.zip › SUPP conversion/toxins-758438 SUPP conversion.pdf]

Supplementary Materials: Expansion and conservation of biosynthetic gene clusters in pathogenic *Pyrenophora* spp.

Paula Moolhuijzen, Mariano Jordi Muria-Gonzalez, Rob Syme, Catherine Rawlinson, Pao Theen See, Caroline S. Moffat and Simon Ellwood

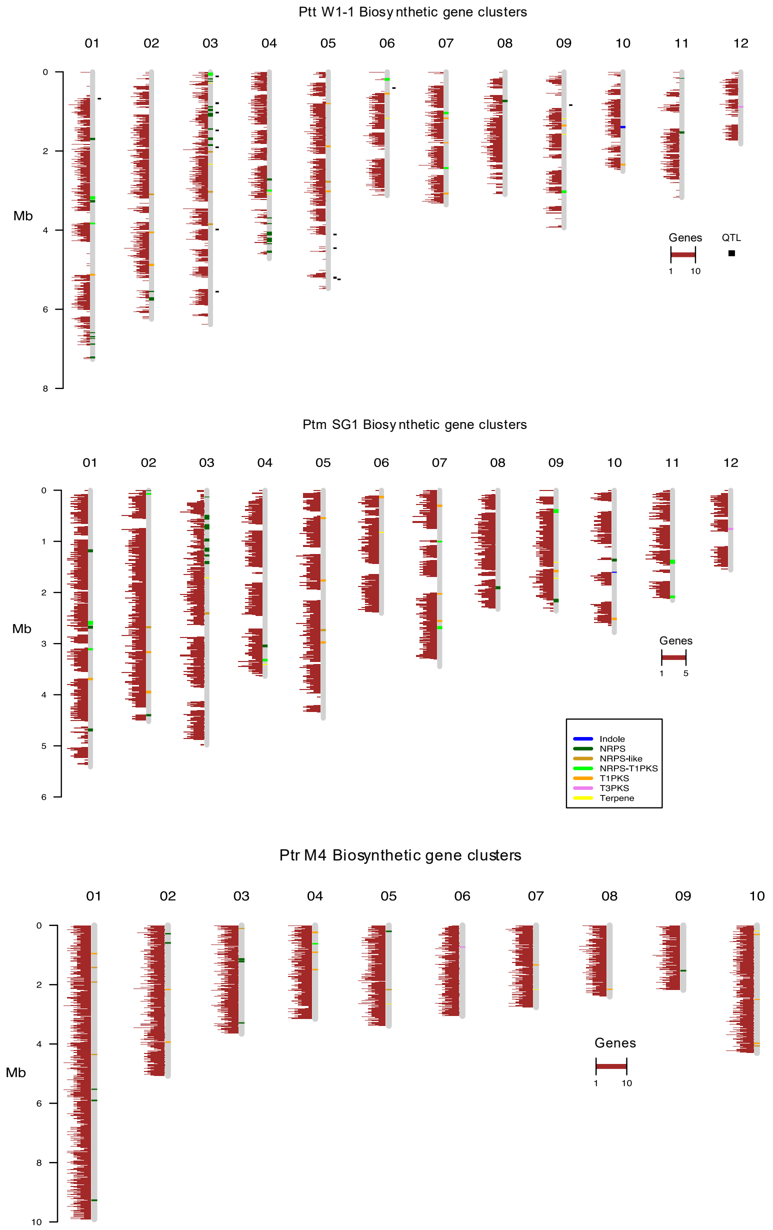

Figure S1. *Pyrenophora* predicted BGC distributions in PttW1-1, Ptm SG1 and Ptr M4 genomes. The gene count (in 10 kb windows) is plotted in red on the left-side of the chromosomes and predicted biosynthetic clusters are shown as NRPS (dark green), T1PKS (orange), terpene (yellow), NRPS-T1PKS (green), T3PKS (violet), NRPS-like (brown) and indole (blue). Virulence QTLs (black) are shown on the right-side of the chromosome for Ptt W1-1.

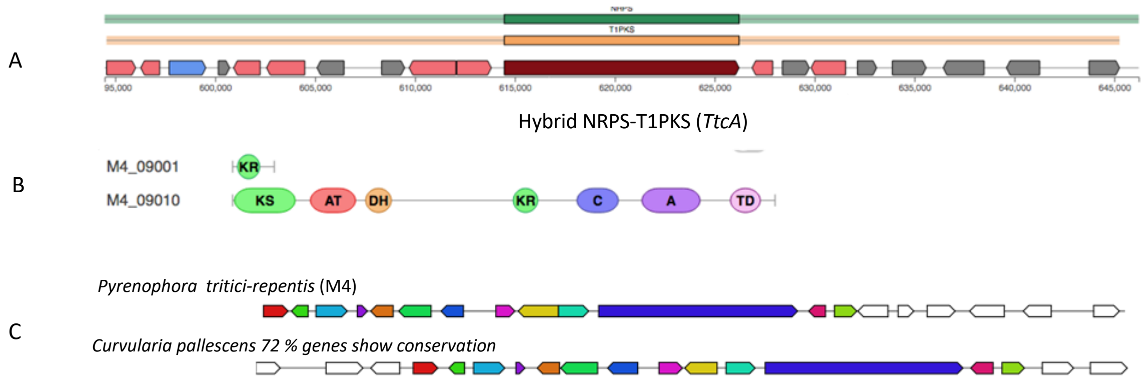

Figure S2. Biosynthetic cluster for triticone production (Ttc cluster) in *P. tritici-repentis* is absent in *P. teres f. teres* and *P. teres f. maculata.* A) Chromosome 4 *P. tritici-repentis* triticone biosynthetic gene cluster (Ttc). B) Domains for core gene M4_09010. C) Biosynthetic gene cluster conservation with *Curvularia pallescens.*
